# Virtual reality-based pain control in endometriosis: a questionnaire-based pilot study of applications for relaxation and physical activity

**DOI:** 10.1007/s00404-025-08000-y

**Published:** 2025-03-25

**Authors:** Viktoria Pakebusch, Barbara Schlisio, Birgitt Schönfisch, Sara Y. Brucker, Bernhard Krämer, Jürgen Andress

**Affiliations:** 1 Department of Obstetrics and Gynecology, University of Tübingen, Calwerstr. 7, 72076 Tübingen, Germany; 2 Department of Anesthesiology and Intensive Care Medicine, University of Tübingen, Hoppe-Seyler-Str. 3, 72076 Tübingen, Germany

**Keywords:** Chronic pain management, Benign gynecological disease, Nonpharmacological treatment, Supportive care, Immersive visual technology

## Abstract

**Purpose:**

Virtual reality (VR) based technology may offer new avenues in the management of chronic endometriosis-related pain. Our prospective, 14-week, open, three-phase, cross-over pilot study investigated whether the use of VR technology equipped with a relaxation-inducing application (VR-R) or an activity-stimulating application (VR-A) could change endometriosis-related chronic pelvic pain levels and impairment of daily life.

**Methods:**

23 women aged 32.7 (SD 8.2) with endometriosis-related pelvic pain were each assigned to a permutated sequence of three 4-week phases: (A) the VR-R, (B) VR-A, and (C) intervention-free control phases. Phases were separated by two interspersed 1-week washout phases. Main outcome measures included: momentary, average, and maximum pain intensities on a 0–10 numerical rating scale (NRS); the Pain Disability Index (PDI) score; the Pain Catastrophizing Scale (PCS) score; sleep quality (Medical Outcomes Study Sleep Scale (MOS-SS) score); the Depression Anxiety Stress Scales (DASS) score; and the general health-related quality-of-life score (Short Form (12) Health Survey (SF-12)).

**Results:**

Compared to baseline, VR-R use showed statistically significant positive effects for several scores (NRS “average pain”; PDI “total score”; PCS “total score” and the “magnification”, “rumination”, and “helplessness” subscores; MOSS-SS “index I and II”; and the DASS “depression” and “stress” subscores), whereas VR-A yielded significant positive changes only for PDI “total score”; PCS “total score” and the “helplessness” and “magnification” subscores; MOSS-SS “index II”; and DASS “depression” and “stress”. As four scale scores also showed significant improvements for control, a comparison of the effects was performed to offset a potential placebo-like effect by comparing difference from baseline against control. This analysis yielded significantly greater positive effects only for VR-R: PCS “total score” and “helplessness”; MOSS-SS “index I” and “index II”; and the three DASS subscores “depression”, “anxiety”, and “stress”. SF-12 showed no significant changes in either analysis.

**Conclusions:**

VR-R and VR-A showed positive effects on several pain and quality-of-life scores, which were significant for some scores compared to baseline. For VR-R, some of these improvements were indeed significantly greater than under control conditions, while the effects with VR-A were not. Larger studies are needed to corroborate these findings.

**Trial registration:**

DRKS00030189.

**Supplementary Information:**

The online version contains supplementary material available at 10.1007/s00404-025-08000-y.

## What does this study add to the clinical work

**Table:** 

A cross-over pilot study of relaxation- or physical activity-inducing virtual reality technology showed improvements in endometriosis-related chronic pain and quality of life using scales to measure pain and other parameters.	

## Introduction

Endometriosis is a common benign but chronic disease in women of reproductive age, in which endometrial cells grow outside the uterine cavity, forming lesions within the uterine wall (adenomyosis) and in extrauterine sites, such as the ovaries, fallopian tubes, and abdominal tissues, including the bladder, intestines, peritoneum, and diaphragm [1–5]. Symptoms, if present, vary greatly from patient to patient [6, 7] and typically include chronic pelvic pain with or without lower back or abdominal pain, dysmenorrhea, dyspareunia, dysuria, dyschezia, ovulation pain (mittelschmerz), and pain during physical activity. As symptoms tend to be unspecific, endometriosis often remains undiagnosed for many months and even up to years [1, 3, 8–10]. Endometriosis-related chronic pain is often severe and can significantly affect the daily life of patients. The resulting medical costs and loss of productivity are also of considerable economic importance due to the frequency of the disease [11–14]. Furthermore, endometriosis can cause infertility [15, 16] and patients with endometriosis often suffer from depression, anxiety and sleep disorders [17].

Treatment options typically include analgesics, surgery, hormonal therapy, and complementary medical treatments [1–3, 18–21]. Nonpharmacologic and nonsurgical approaches to the management of chronic pain include psychotherapy, distraction techniques, and physical activity [22].

With the advent of virtual reality (VR) based technology in recent years, new avenues are currently also being explored in the management of chronic endometriosis pain [23, 24]. More generally, VR has been shown to be effective in the treatment of chronic pain associated with other diseases, or in the treatment of comorbidities such as anxiety and depression, as recent meta-analyses have shown [25, 26].

Against this backdrop, we sought to investigate the potential beneficial effects of using VR headsets equipped with relaxation (HypnoVR^®^) and activity (SyncVR Fit^®^) applications on self-reported endometriosis-related chronic pain, pain-related disability in everyday life, health-related and overall quality of life, and the general well-being of endometriosis patients.

## Methods

### Study population

Eligible for inclusion were women aged at least 18 years with a histologically confirmed diagnosis of endometriosis, reporting endometriosis-related severe pelvic pain with pre-study baseline ratings of at least 5 points on visual numerical rating scale (NRS). Recruitment occurred during routine outpatient visits to our university level-3 endometriosis center. Baseline ratings were derived from patient-reported NRS ratings for the preceding four weeks. Further eligibility criteria included the willingness not to have surgery or begin a new drug or nondrug treatment (opioid or nonopioid analgesic, acupuncture, behavioral therapy, massages, etc.) during study participation. Inclusion also required an adequate knowledge of German and the assurance that the study devices would be used according to manufacturers’ instructions.

Exclusion criteria included serious mental illness (e.g., psychosis, schizophrenia, or bipolar disorder) and major depression based on screening with the aid of the study questionnaires and the Depression Anxiety Stress Scales (DASS). Starting regular use of opioid analgesics within seven days preceding study entry was also an exclusion criterion.

### Study objectives

The aim of this study was to investigate whether the use of VR technology with a relaxation application (HypnoVR^®^) or an activity application (SyncVR Fit^®^) had an effect on the reported pain level and endometriosis pain-related impairment of daily life.

Primarily, we investigated momentary, average, and maximum pain intensity per day. Secondary objectives included recording (1) pain-related disability as measured using the Pain Disability Index (PDI); (2) sleep quality as measured using the Medical Outcome Study–Sleep Scale (MOS-SS); and (3) general health-related quality of life as measured using the 12-item Short Form (12) Health Survey (SF-12).

### Study design, devices, and interventions

*Study design. *This study was a prospective, 14-week, open, three-phase, cross-over pilot study with 1-week washout periods interspersed between phases. Each participant sequentially completed a four-week intervention phase with each of the two study devices, and a four-week intervention-free control phase. The sequence of the two intervention phases and the control phase was permutated, yielding an allocation plan with six different sequences. Participants were consecutively included and assigned to one of the six predetermined intervention/control sequences in accordance with the allocation plan. Questionnaires were completed at baseline and after each study phase. Additionally, participants were provided with a paper-based pain diary for daily completion (see below). Participants had four interviews, one at the initial visit (eligibility assessment and baseline data collection) and one after completing each study phase.

The study protocol received prior approval from the ethics committee of the medical faculty of Tübingen University Hospital, Tübingen, Germany (approval number 893/2021B01) in accordance with the ICH-GCP guidelines, the Declaration of Helsinki, and all relevant laws and regulations. All participants gave their prior written informed consent.

**Fig. 1:**
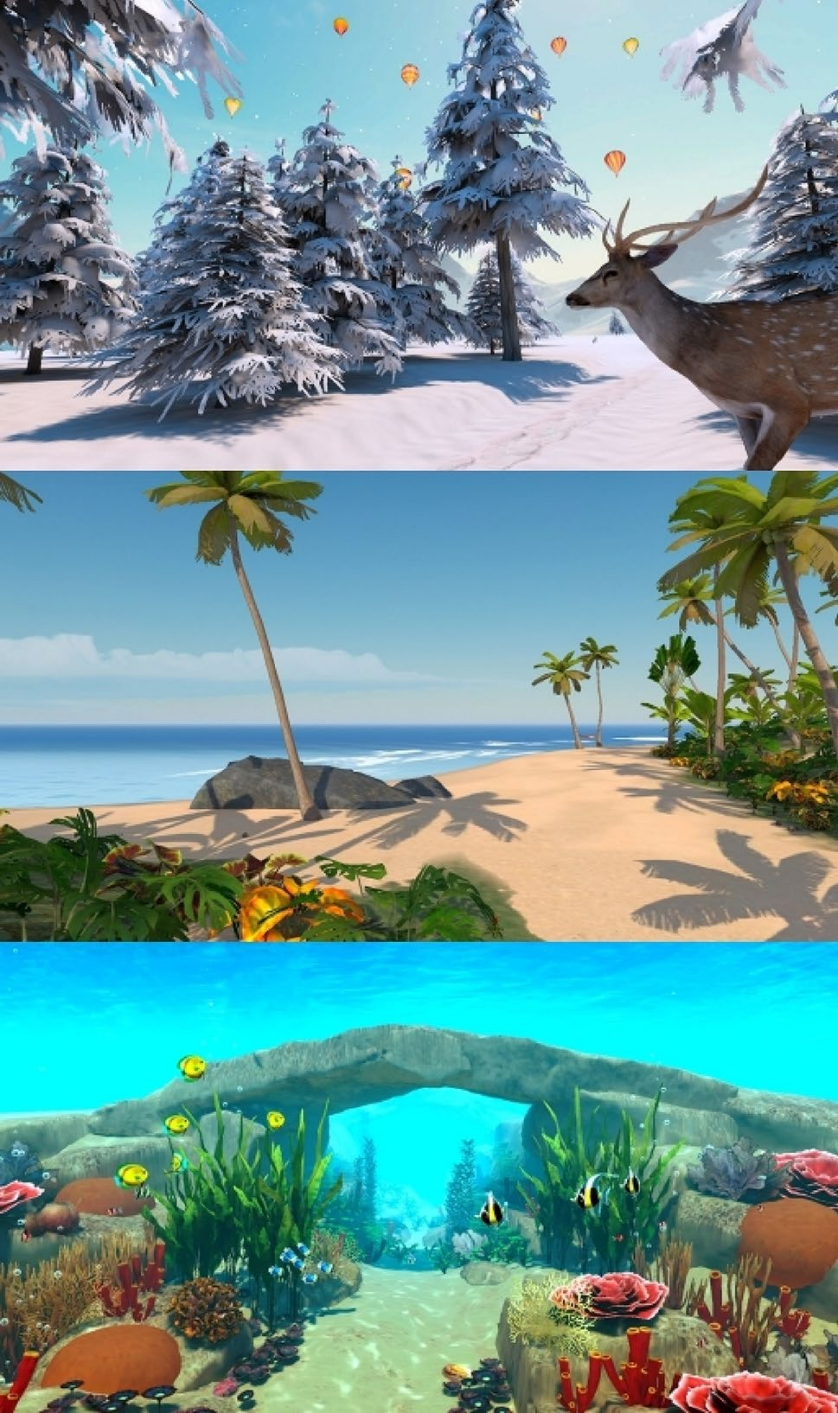
Examples of 3D-immersive virtual reality environments for relaxation (VR-R): a winter day (top), an ocean beach (middle), and an underwater landscape (bottom) (reproduced with kind permission from the rights owner)

**Fig. 2:**
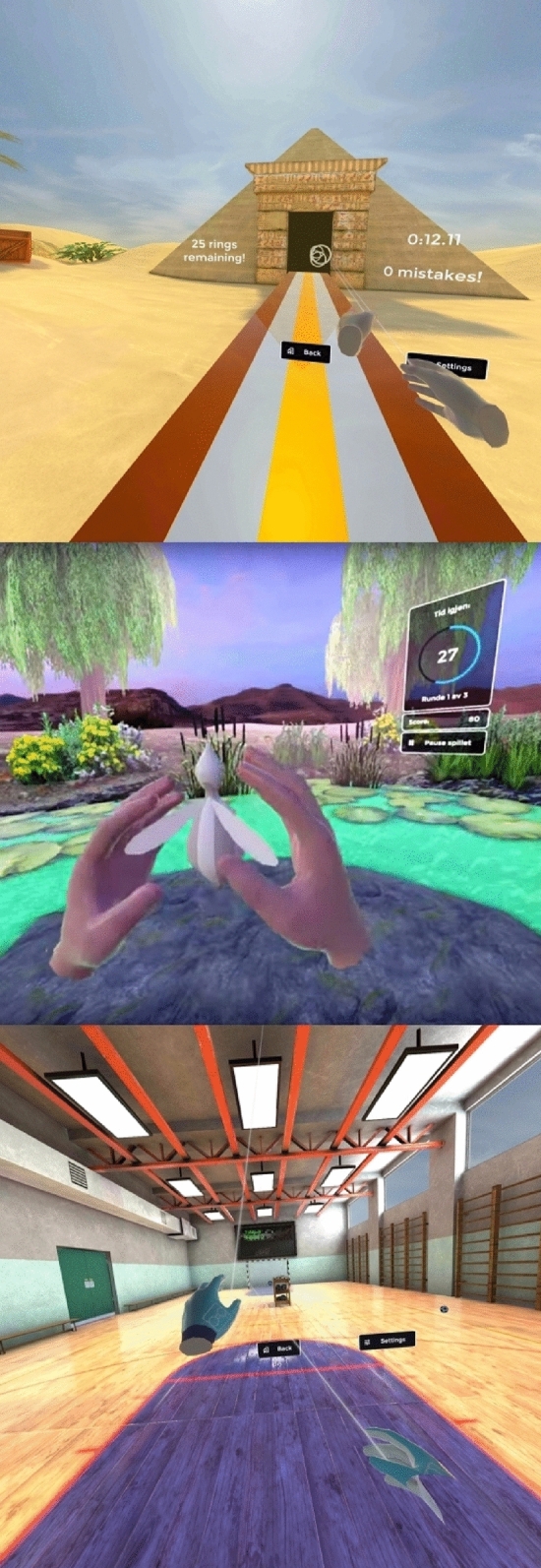
Examples of 3D-immersive virtual reality environments inducing physical activity (VR-A): throwing rings into a pyramid (top), catching fireflies (middle), and throwing soccer balls (bottom) (reproduced with kind permission from the rights owner)

*Study devices. *Two virtual reality (VR) headsets were used as study devices: the Pico G2 4 K (Pico Technology Co., Ltd, Beijing, China) with the pre-installed HypnoVR^®^ (HypnoVR SAS, Lampertheim, France, www.hypnovr.io/en) application and the Pico Neo 3 Pro with the pre-installed SyncVR Fit^®^ (Amersfoort, The Netherlands, www.syncvrmedical.com) application. Virtual experiences with the HypnoVR^®^ included, e.g., a journey through space and a deep-sea dive for virtual reality-induced relaxation (VR-R), whereas the SyncVR Fit^®^ offered virtual reality-induced activity (VR-A) by motivating participants to be physically active and, e.g., perform guided body movements or play soccer in a virtual stadium. Examples of the 3D-immersive VR environments provided by the VR-R and VR-A applications are shown in Figs. 1 and 2.

*Study interventions. *These consisted in using the assigned VR device for 15–20 min three times a week, completing the relevant online questionnaires described below, and documenting the experience in an electronic pain diary online. In addition, *pro re nata* (PRN, as needed) use was permitted, but needed to be recorded. During the control phase, participants were required to complete the questionnaires and keep their pain diary.

### Data collection

*Questionnaires. *Table 1 lists the questionnaires used to record basic demographic and medication-related data as well as three pain-related questionnaires and three questionnaires pertaining to quality of sleep, quality of life, and mental health. Participants were requested to complete the questionnaires at the same time of day (7 pm, if possible).

**Table 1:** Study questionnaires

Questionnaire	Purpose
Basic questionnaire	Collection of demographic data, including age, weight, height, previous and current analgesic (long-term or PRN) medication
Numerical rating scale (NRS) for pain	Subjective assessment of pain intensity on a 0–10 rating scale
Pain disability index (PDI)	Self-assessment of chronic pain-related disruption of everyday life activities to measure subjective pain-related disability
Pain catastrophizing scale (PCS)	Measurement of exaggerated negative perception of pain, comprising 3 subscales (“magnification”, “rumination”, and “helplessness”)
Medical Outcome Study–Sleep Scale (MOS-SS)	Assessment of the quality and possible impairment of night sleep
Depression Anxiety and Stress Scale (DASS)	Determination of the probability of the presence of depressive disorder or anxiety disorder
Short Form (12) Health Survey (SF-12)	12-item self-reported survey of general health-related quality of life

Decreases in scores indicate improvement except with SF-12, where increases indicate improvement

*PRN pro re nata* (as needed)

Participants used a secure web-based survey platform (Unipark^®^, Tivian XI GmbH, Cologne, Germany; www.unipark.com) hosting the NRS, PDI, PCS, MOS-SS, DASS, and SF-12 questionnaires to record their pain ratings, additional self-assessments, and use of pain medication. No personal data were stored on the Unipark^®^ platform.

*Pain diary. *Participants were instructed to keep a purpose-designed, paper-based pain diary throughout the study duration (including washout periods), detailing momentary pain at rest, average pain, and maximum pain intensity at the same time of day (7 pm, if possible). They were also requested to provide information on their medication requirements, including PRN medication, and their menstrual cycle, including typical cycle duration, duration of menstruation, and whether they had experienced menstrual bleeding or spotting within the last 24 h.

### Data analysis

Statistical analysis was performed using R, Version 4.2.2. Descriptive statistics used numbers and percentages and means and standard deviations (SD). The difference to baseline was assessed by paired Wilcoxon–Mann–Whitney rank test. To investigate the effects of treatments, the differences to baseline Δ_B_ for VR-R and VR-A were compared versus the Δ_B_ for control. A linear model for PRN medication depending on the presence or absence of menstrual bleeding was formulated with participant as random factor. A significance level of 5% was chosen in all statistical tests.

## Results

### Study population

The study was conducted between 10/2022 and 02/2023. Out of 25 eligible candidates meeting the inclusion criteria, 2 discontinued study participation after completing the baseline visit and the baseline questionnaire and were therefore excluded from the study. Table 2 summarizes the 23 participants’ baseline demographic and clinical characteristics.

**Table 2:** Baseline demographic and clinical characteristics of the study population (n = 23)

	Mean (SD) or number | percentage
Demographics	
Age at baseline^*^, years	32.7 (8.2)
Body mass index, kg/m^2^	22.6 (4.4)
Endometriosis	
Duration of endometriosis-related pain, years	
< 1	0 | 0%
1–5	7 |30%
> 5	16 |70%
Comorbidities (multiple entries possible)	
Anxiety disorder	0|0%
Chronic lower back pain	2|9%
Chronic headaches	3|13%
Depression	3|13%
Other pain disorders	7|30%
No comorbidities	12|52%
Pain medication	
Opioids	3|13%
Before study entry	3|13%
After study entry	3|13%
Nonopioids	21|91%
Daily use	3|13%
Occasional use	18|78%
Hormone treatment	12|52%
Oral contraceptive	9|39%
Hormonal intrauterine device	2|9%
Vaginal ring	1|4%

*SD* standard deviation

*Age is given as patient-reported full years

Participants stated their occupations as: homemaker (1/23), student or apprentice (6/23), job seeker (1/23), full-time worker (10/23), or part-time worker (5/23). None indicated being unable to work.

Endometriosis-related pain had persisted for 1 to more than 5 years in all participants at study initiation, with 11 of 23 (48%) participants reporting comorbidities. Single instances of other complaints reported by 12 of 23 (52%) included cold symptoms, hormone fluctuations, exhaustion/fatigue, gastrointestinal complaints (bloating, pain, food intolerance), headaches, sleep disorders, fears, palpitations, leg cramps, frequent urge to urinate, back pain, dyspareunia, and hypothermia, amongst others.

Treatment with hormones as contraceptives or endometriosis medications was reported by 12 out of 23 (52%). Reports of nonpharmacologic treatments for pain included physiotherapy (5 of 23 patients), psychotherapy (3 of 23), and acupuncture and relaxation techniques (5 of 23).

### Completeness of data sets

Two participants did not complete the baseline questionnaire. One participant discontinued after the first phase and another after the second phase. Pain diaries were missing for two other participants. Due to the small number of participants and since this was a pilot study, these participants were not excluded.

### Pain diary

*Pain diary. *Complete pain diaries were obtained from 21 out of 23 (91%) participants. Scatter diagram analysis of the questionnaire and pain diary recordings of average and maximum pain matched well (with respective Pearson correlation coefficients of r_P_ = 0.84 and r_P_ = 0.90). VR-A and VR-R phases were not associated with any significant differences in diary-recorded pain scores averaged per participant when compared against control or each other.

*Menstrual bleeding. *Momentary pain at rest, average pain, and maximum pain averaged per participant were all significantly greater with menstrual bleeding than without (p = 0.027, p = 0.011, and p = 0.008, respectively).

*PRN medication. *There was no significant difference in PRN medication averaged per participant in the presence or absence of menstrual bleeding (p = 0.114). A linear model for PRN medication depending on the presence or absence of bleeding with participant as random factor showed a significant difference (p < 0.001). These results demonstrate the importance of collecting menstrual cycle data.

**Fig. 3:**
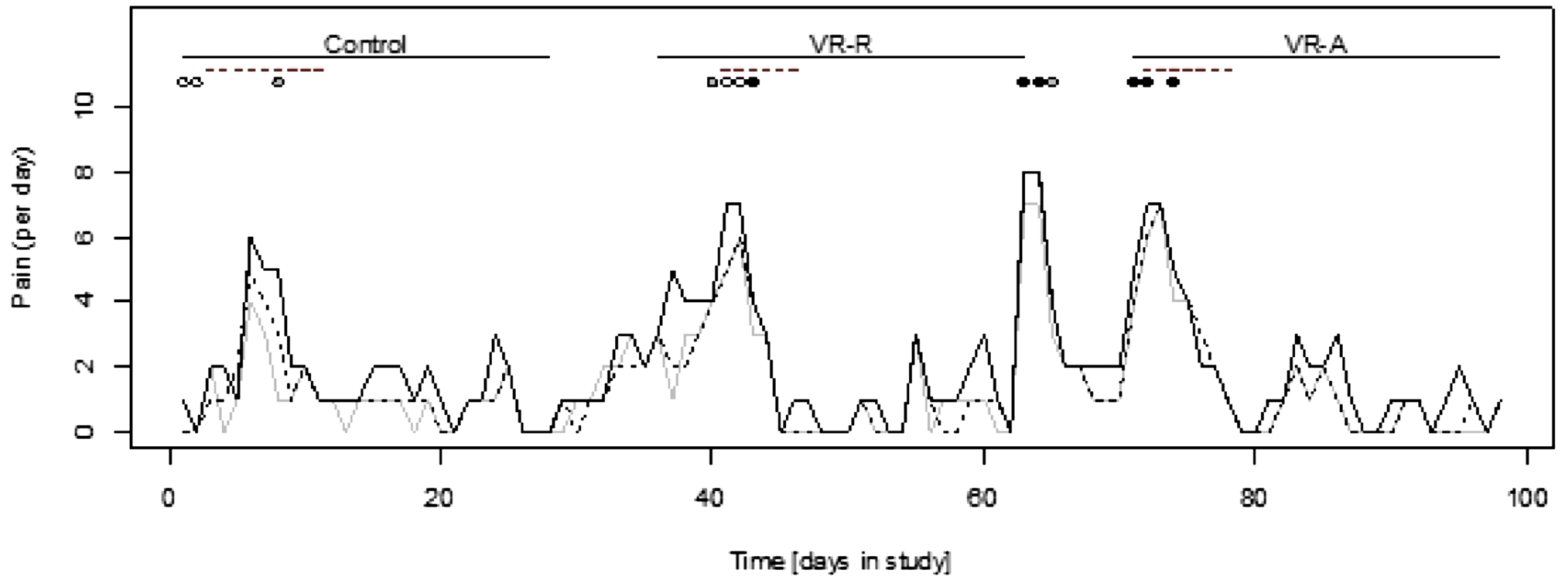
Example of a time course of participant-reported pain ratings in the diary throughout the study. Solid black line = maximum pain; dashed line = average pain; solid grey line = momentary pain; red dashes = days with menstrual bleeding; circles = PRN medications

Figure 3 shows an example of the time course of pain ratings as reported by one participant during the study. Generally, pain ratings in all three pain categories increased with the onset of menstruation, accompanied by increased consumption of PRN medication.

### Questionnaires

#### Pain numerical rating scale

**Fig. 4:**
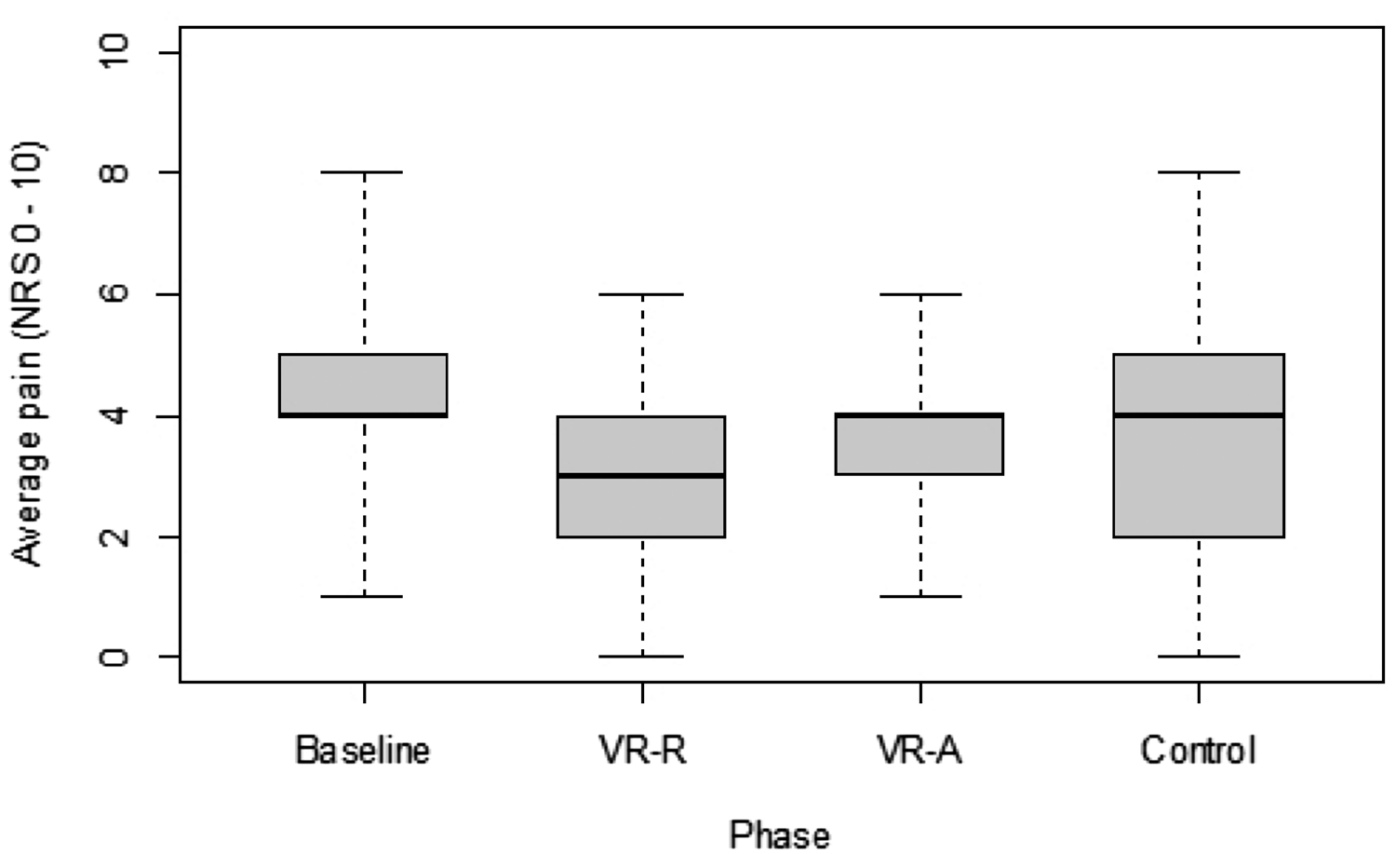
Average pain on the NRS scale as reported for baseline, virtual reality-guided activity (VR-A) and relaxation (VR-R), and control

The pain NRS questionnaire measures momentary pain severity and average and maximum pain during the preceding 4 weeks. Figure 4 shows that for average pain, the mean NRS value of 3.3 exhibited a significant difference versus baseline (4.4) for VR-R (p = 0.033), whereas VR-A (3.6) and control (3.8) did not. No significant differences in NRS scores versus baseline were observed for momentary pain and maximum pain (data not shown). Comparisons of differences from baseline Δ_B_ yielded no statistically significant effects for momentary, average, and maximum pain on the NRS scale observed with either VR-A and VR-R versus control (Table 3).

**Table 3:** Qualitative summary of questionnaire results for virtual reality interventions and control versus baseline, and changes from baseline Δ_B_ for virtual reality interventions versus control

	VR-R vs. baseline n = 22	VR-A vs. baseline n = 21	Control vs. baseline n = 23	Change from baseline Δ_B_	
				VR-R vs. control n = 22	VR-A vs. control n = 21
Pain on NRS scale (↓ = improvement)					
Momentary pain	↓	↓	↓	NS	NS
Average pain	↓*	↓	↓	NS	NS
Maximum pain	↓	→	↓	NS	NS
PDI (↓ = improvement)					
Total score	↓*	↓*	↓*	NS	NS
PCS (↓ = improvement)					
Total score	↓*	↓*	↓*	Significant	NS
Helplessness	↓*	↓*	↓	Significant	NS
Magnification	↓*	↓*	↓*	NS	NS
Rumination	↓*	↓	↓*	NS	NS
MOSS-SS (↓ = improvement)					
(Sleep) Index I	↓*	↓	↓	Significant	NS
(Sleep) Index II	↓*	↓*	↓	Significant	NS
DASS (↓ is improvement)					
Depression	↓*	↓*	↓	Significant	NS
Anxiety	↓	↓	↑	Significant	NS
Stress	↓*	↓*	↓	Significant	NS
SF-12 (↑ = improvement)					
Physical health	↑	↑	↑	NS	NS
Mental health	↑	↑	↓	NS	NS

*NS* not significant, *NRS* numerical rating scale, *PDI* pain disability index, *PCS* pain catastrophizing scale, *MOSS-SS* medical outcome study – sleep scale, *DASS* depression anxiety and stress scale, *SF-12* short form (12) health survey SF-12, *VR-R* VR relaxation, *VR-A* VR activity

*Significant increase or decrease in measured scores

#### Pain disability index (PDI)

The 7-item Pain Disability Index (PDI) questionnaire measures self-reported pain-related impairment [27]. PDI scores were significantly lower than baseline for VR-A, VR-R, and control (p = 0.008, p = 0.003, and p = 0.008, respectively). Testing Δ_B_ values of VR-A and VR-R versus control yielded no statistically significant differences (p = 0.270 and p = 0.457, respectively).

#### Pain catastrophizing scale (PCS)

The 13-item Pain Catastrophizing Scale (PCS) measures the extent of pain catastrophizing, distinguishing 3 subscales: “rumination”, “magnification,” and “helplessness” [28, 29]. PCS total scores and all three PCS subscores for both VR applications differed significantly from baseline, as did the control, with the exception of the “helplessness” subscore for the control and the “rumination” subscore for VR-A.

However, comparison of the differences from baseline (Δ_B_ values) for the PCS total score and the PCS subscores of VR-A and VR-R against control showed no statistically significant effects, except for the PCS total score (p = 0.043) and the PCS “helplessness” subscore (p = 0.023) observed with VR-R versus control.

#### Medical outcomes study sleep scale (MOS-SS)

The 12-item MOS-SS questionnaire measures sleep quality during the 4 weeks before questioning [30]. MOS-SS questionnaire items are detailed in Supplementary Table 1 online (based on [31]). MOS-SS scores for sleep disturbance differed significantly from baseline for VR-A, VR-R, and control. For VR-R, the scores for somnolence, index I, and index II also differed significantly from baseline; for VR-A, the index II score differed significantly from baseline. Testing Δ_B_ values of VR-A and VR-R versus control yielded significant effects only for the index I and index II scores (p = 0.019 and p = 0.003, respectively).

#### Depression anxiety stress scale (DASS)

The 21-item DASS self-report questionnaire comprises 7 questions per subscale to assess signs of depression (DASS-D), anxiety (DASS-A), and stress (DASS-S) over the past week [32, 33]. DASS -D and DASS-S subscores were statistically significantly different from baseline for both VR applications but not for control, whereas the DASS-A subscores were not significantly different. By contrast, testing differences from baseline (Δ_B_ values) VR-R versus control yielded significant effects for all 3 subscores (p = 0.014 for DASS-D, p = 0.033 for DASS-A, and p = 0.004 for DASS-S).

#### SF-12 health survey

The Short Form (12) Health Survey is a 12-item, self-reported health survey that provides physical health and mental health scores [34]. In our study, neither VR application yielded statistically significant results, whether comparisons were performed as scores versus baseline or Δ_B_ versus control.

### Summary of results

Overall, the results of our 14-week VR-based intervention study of pain disability; sleep; depression, anxiety, and stress; and physical and mental health demonstrates that, compared versus baseline, both VR applications showed statistically significant reductions in some of the scores for the respective rating scales employed. Significant differences were also seen for the control phase and baseline (Table 3).

## Discussion

### Principal findings

Endometriosis is often associated with severe chronic pain. Patients with chronic pain tend to feel helpless and dependent on others, which in turn may trigger, inter alia, depression and anxiety [17]. Current guideline-based standard pain treatments involving analgesics, hormone therapy, and surgery are frequently inadequate and may entail specific risks and considerable side effects. Supportive care also provides only limited relief and is often unsatisfactory [22]. Hence there is a need for new methods that are readily available, have few side effects, and will help to restore patients’ sense of self-efficacy.

With the advent of VR technology, which immerses the user in an alternative audio-visual reality, VR-based distraction, relaxation, and physical activity interventions have been found to be effective in the treatment of chronic pain of various etiologies [25, 26]. The present study investigated the use of virtual reality applications in endometriosis patients with regard to pain, sleep quality, mental health, and quality of life. To our knowledge, our study was the first to compare the effects of two different VR applications, i.e., relaxation (VR-R) and activity (VR-A), against a VR intervention-free control phase in a cross-over design. Moreover, it was also the first study to run over a longer period of 14 weeks in total to investigate medium-term effects on the above-mentioned areas investigated.

Our study showed that the use of VR-R led to significant improvements in several pain and quality-of-life scores. As improvements were also seen in the control phase, in contrast to other studies [23, 24], we performed further analyses which took into account the change from baseline Δ_B_ in order to determine as precisely as possible the effect of the intervention as such, so as to differentiate the effect of the VR intervention from the effect of study participation (potential placebo-like effect in the control).

With VR-R, changes that were significantly greater than control were thus observed for the PCS (“Total score” and the “helplessness” subscore), MOSS-SS (“index I” and “index II”), and DASS (“depression”, “anxiety”, and “stress”) scores. Positive effects were also observed for “average pain”, PDI “total score”, and the PCS “magnification” and “rumination” subscores, although these were not significant when compared to the changes in the control. With VR-A, improvements from baseline were noted for the PDI (“total score”), PCS (“total score”, “helplessness”, and “magnification”), MOSS-SS (“index II”), DASS (“depression” and “stress”) scores, but these were not significantly greater than the effects in the control.

A comparison of the present study with previously published data reveals clear differences. In 2022, Merlot et al. [23] published the results of a randomized controlled trial in 45 women with endometriosis-related chronic pelvic pain. They investigated a single immersion-based VR intervention for pain treatment, followed by 6 pain recordings over a period of 4 h. By contrast, we recorded 84 pain scores and quality-of-life data items per participant in a crossover design over a 14-week study period.

A second study by Merlot et al. published in 2023 [24] revisited the topic, expanding the study to 102 participants treated with immersive VR technology after repeated use of VR, but again limited to 2–5 days starting from menstruation. The total of 4 measurements of pain perception were also only taken around the intervention on the same day. Of particular note, the authors did not account for the potential placebo-like effect in the control group, which in contrast to our study was a “control group” with intervention but without immersive technology. Nevertheless, the authors speak of a significant improvement in pain perception through the use of VR with immersive technology. By contrast, our present study attempted to analyze the efficacy of both VR-R and VR-A as precisely as possible by addressing the potential influence of mere participation in the study.

### Strengths and limitations

Several strengths and limitations of our study should be considered when interpreting our results. One strength is that this was the first study to compare the effects of 4-week periods of VR-based relaxation and VR-based exercise therapy on pain and quality-of-life related parameters with a 4-week VR intervention-free control phase. Also, the crossover design enabled us to conduct this pilot study with a comparatively small number of participants. Furthermore, although treatment allocation was not randomized in the strict sense, the preestablished allocation table eliminated arbitrary assignment of consecutive patients to a specific treatment sequence. Carry-over effects were minimized by 1-week washout periods in between study phases. Of note, compliance with study requirements was very high across all participants, which was reflected not least in the high quality of the data. Moreover, the pain diary entries matched closely with the questionnaire responses. In addition, while favoring VR-based relaxation, all participants stated they had subjectively benefited from the study. Last, our study population was balanced with regard to hormone therapy, with half of participants (52%) receiving hormones.

A specific, and likely the most important limitation of the study was its small sample size of 23 participants, which may have impacted the significance of the results and therefore warrants special consideration in future, larger studies. However, this was a pilot study with a cross-over design, which allowed us to keep the sample size to a minimum. Further sample-size considerations included the logistics of exchanging the high-cost devices between participants in the midst of the ongoing COVID-19 pandemic. In this context it is remarkable that only two of the 23 participants discontinued their participation. Due to the small sample size, it was not possible to take the sequence of study phases into account in the analysis. However, our findings show the importance of varying the sequence of study phases, ideally to use all possible permutations equally often in the absence of randomization.

## Conclusions

VR-R was shown to have positive effects on a number of pain and quality-of-life scores, which were significant for several scores when compared to baseline, and some of these effects were even shown to be significantly larger than those observed in the control. Moreover, VR-A showed significant positive effects compared to baseline; however, in our small sample, these effects were not significantly greater than those in the control. We conclude that VR-R may have a greater impact than VR-A but concede that both VR options need to be further explored in larger studies.

## Supplementary Information

Below is the link to the electronic supplementary material.

Supplementary file1 (PDF 133 KB)

## Data Availability

No datasets were generated or analysed during the current study.

## Abbreviations

DASS: Depression anxiety and stress scale
MOS-SS: Medical outcome study-sleep scale
NRS: Numerical rating scale
NS: Not significant
PCS: Pain catastrophizing scale
PDI: Pain disability index
PRN: Pro re nata (as needed)
SF-12: Short form health survey SF-12
VR: Virtual realty
VR-A: VR activity
VR-R: VR relaxation
