# Supplementary material for: Virtual reality-based pain control in endometriosis: a questionnaire-based pilot study of applications for relaxation and physical activity
Source: Arch Gynecol Obstet. 2025 Mar 25;311(6):1721–31. doi: 10.1007/s00404-025-08000-y (PMC12055868; doi:10.1007/s00404-025-08000-y)
Supplement: Supplementary file 1 — Supplementary file1 (PDF 133 KB) [file 404_2025_8000_MOESM1_ESM.pdf]

Supplementary material

Supplementary table 1. Items on the MOS-SS questionnaire

(modified from Viala-Danten et al. (2008) [31])

Dimensions and Sleep Problems Index	Item
**Sleep disturbance**	
Trouble falling asleep	07
Sleep restlessness	03
Awaken during sleep	08
Time to fall asleep	01
**Somnolence**	
Trouble staying awake	09
Take naps	11
Feel drowsy	06
**Sleep adequacy**	
Enough sleep, feel rested	04
Amount sleep needed	12
**Snoring**	
Snore during sleep	10
**Awaken short of breath or headache**	
Awaken short of breath or headache	05
**Quantity of sleep/Optimal sleep**	
Quantity of sleep	02
**Sleep Problems Index I**	04 Enough sleep, feel rested, 05 Awaken short of breath or headache, 07 Trouble falling asleep, 08 Awaken during sleep, 09 Trouble staying awake, 12 Amount sleep needed
**Sleep Problems Index II**	01 Time to fall asleep, 03 Sleep restlessness, 04 Enough sleep, feel rested, 05 Awaken short of breath or headache, 06 Feel drowsy, 07 Trouble falling asleep, 08 Awaken during sleep, 09 Trouble staying awake, 12 Amount sleep needed
